# Rumination and subjective well-being: The chain mediating role of emotion regulation difficulties and problematic social media use

**DOI:** 10.1371/journal.pone.0353931

**Published:** 2026-07-16

**Authors:** Huan Song, Yueyu Shu, Ping Ren

**Affiliations:** 1 School of Psychology, Northwest Normal University, Lanzhou, China; 2 School of Educational Science, Neijiang Normal University, Neijiang, China; 3 Faculty of Elementary Education, Nanchong Vocational College of Culture and Tourism, Nanchong, China; South China Normal University, CHINA

## Abstract

**Objectives:**

Based on the Compensatory Internet Use Model and the I-PACE framework, the present study develops a serial mediation model grounded in a cognition-emotion-behavior pathway. Specifically, this study examined whether difficulties in emotion regulation and problematic social media use were associated with the relationship between rumination and subjective well-being among a sample of vocational college students within the context of increasingly digitalized everyday life.

**Methods:**

A survey was conducted among 402 vocational college students using the Ruminative Responses Scale, the Brief the Version of the Difficulties in Emotion Regulation Scale, the Problematic Social Media Use Questionnaire, the Satisfaction with Life Scale and the Positive and Negative Affect Schedule.

**Results:**

Rumination was positively associated with difficulties in emotion regulation and problematic social media use, whereas subjective well-being was negatively associated with all three variables. Difficulties in emotion regulation were also positively correlated with problematic social media use. Mediation analyses identified three significant indirect pathways: the mediating role of difficulties in emotion regulation, accounting for 35.49% of the total effect; the mediating role of problematic social media use, accounting for 22.35%; and the serial mediating role of difficulties in emotion regulation and problematic social media use, accounting for 12.16%.

**Conclusions:**

The findings suggest that difficulties in emotion regulation and problematic social media use are associated with the relationship between rumination and subjective well-being among the sampled vocational college students. These findings may provide preliminary implications for interventions targeting emotion regulation and problematic social media use among vocational college students.

## Introduction

Subjective well-being refers to individuals’ cognitive evaluations and emotional experiences of their own lives [1], and serves as a core indicator for measuring mental health and social adjustment. Vocational college students, as an important group characterized by both vocational and developmental features, are in a critical transitional stage from late adolescence to early adulthood. Their subjective well-being is closely related to academic engagement, mental health, and social adaptation. Compared with students in traditional undergraduate institutions, vocational college students may experience higher levels of employment pressure, greater uncertainty regarding career development, and relatively limited social support resources, all of which may increase their vulnerability to negative emotional experiences and psychological adjustment difficulties. At the same time, social media has become an important means for vocational college students to regulate emotions, relieve stress, and maintain social connections. From the perspective of compensatory internet use, under conditions of elevated psychological stress and emotional vulnerability, this population may be more inclined to rely on social media to alleviate negative emotions, thereby showing stronger potential associations among rumination, emotion regulation difficulties, and problematic social media use. Therefore, focusing on vocational college students may help clarify the potential mechanisms through which cognitive, affective, and behavioral factors jointly influence subjective well-being, and may also provide implications for mental health interventions in this population.

Individual subjective well-being is influenced by a range of psychological factors. Among these, rumination, defined as a repetitive and passive cognitive processing style focused on negative experiences and emotions [2,3], has received considerable attention in research on college students. When facing academic setbacks, interpersonal conflicts, and identity-related challenges, vocational college students may be more likely to engage in repetitive negative thinking while lacking effective problem-solving and coping strategies.

Existing studies have confirmed that rumination is significantly associated with decreased well-being [4,5] and can negatively associated with students’ subjective well-being [6,7]. However, the association between rumination and well-being may not be simple or direct, and may be related to emotional and behavioral factors within the digital context. Although previous research has examined the association between these variables, the potential emotional and behavioral pathways underlying this association in the digital context remain unclear.

According to cognitive behavioral theory, rumination may lead individuals to persistently focus on negative cognitions and emotional experiences, which may interfere with their ability to identify, understand, and accept emotions, thereby contributing to greater difficulties in emotion regulation [8]. Difficulties in emotion regulation refer to persistent problems in emotional awareness, understanding, acceptance, and use of adaptive regulation strategies [9]. Previous studies have shown that individuals with higher levels of rumination tend to report greater difficulties in emotion regulation [10,11], whereas difficulties in emotion regulation are negatively associated with subjective well-being [12]. Therefore, difficulties in emotion regulation may play an important mediating role between rumination and subjective well-being.

Problematic social media use may represent an important behavioral mechanism linking rumination and subjective well-being. Problematic social media use refers to excessive and maladaptive engagement with social media, which has been associated with adverse physiological, psychological, and behavioral outcomes [13]. According to the compensatory internet use theory, when individuals are unable to effectively cope with internal distress, such as persistent negative emotions associated with rumination, vocational college students may become more likely to use social media as a means of stress avoidance and emotional compensation, thereby obtaining temporary psychological relief [14,15]. Previous studies have shown that rumination is positively associated with problematic social media use [16–18], whereas problematic social media use is negatively associated with subjective well-being [19]. Therefore, problematic social media use may play a mediating role between rumination and subjective well-being.

Building on this, the I-PACE model provides an important theoretical framework for explaining the cognitive, affective, and behavioral processes underlying problematic internet-related behaviors [20]. The model proposes that relatively stable cognitive tendencies may influence emotion regulation processes and further relate to problematic social media use behaviors. Symptom rumination and compulsive thinking can be viewed as maladaptive states of cognitive control dysregulation, characterized by repetitive focus on negative experiences and distressing information. Such persistent cognitive engagement may interfere with adaptive emotion regulation processes, including emotional awareness, emotional acceptance, and cognitive reappraisal [9]. Consequently, individuals with higher levels of rumination are more likely to become trapped in negative emotional states and experience difficulty disengaging from emotional distress, thereby exhibiting greater emotion regulation difficulties [10,11]. Consistent with the I-PACE model’s emphasis on compensatory behaviors, individuals with greater emotion regulation difficulties may be more inclined to rely on external means to rapidly alleviate negative emotions. Due to the accessibility, immediate reward properties, and attentional appeal of social media, it may easily become a frequently used external emotion regulation strategy. Indeed, previous studies have found that emotion regulation difficulties are significantly positively associated with problematic social media use [21,22]. Taken together, rumination as a stable cognitive tendency may contribute to problematic social media use through increased emotion regulation difficulties. In this sense, emotion regulation difficulties and problematic social media use may represent affective and behavioral pathways, respectively, linking cognitive vulnerability to subjective well-being.

Accordingly, the present study focused on vocational college students and examined a serial mediation model linking rumination, difficulties in emotion regulation, problematic social media use, and subjective well-being. It aims to examine the psychological associations among rumination, difficulties in emotion regulation, problematic social media use, and subjective well-being among vocational college students, and to provide theoretical insights and practical implications for promoting well-being through emotion regulation and healthier social media use.

## Methods

### Participants

In this study, Monte Carlo Power Analysis for Indirect Effects was utilized to calculate the sample size. The following parameters were entered: Model = Two Serial Mediators, Power = 0.8, and Confidence Level = 99%. Additionally, the correlations and standard deviation between the variables were inputted. The results indicated that a sample size of 230 would achieve an effect size of 0.8.

A total of 435 vocational students from a higher vocational college in Sichuan Province, China, were recruited through convenience sampling between December 11 and December 28, 2025. Data were collected via an online survey platform (https://www.wjx.cn/). Before completing the questionnaire, participants were informed of the study purpose and provided written informed consent. They were also encouraged to respond honestly and anonymously. To ensure data validity, attention-check items requiring predetermined responses were embedded in the questionnaire, and participants who failed these checks were excluded from subsequent analyses. Questionnaires with missing responses or completion times exceeding three standard deviations from the mean were also excluded as invalid. A total of 402 valid questionnaires were collected, resulting in a response rate of 92.41%. Among the participants, 105 participants were aged 18 years or younger (26.12%), 198 were aged 19 years (49.25%), 75 were aged 20 years (18.66%), and 24 were aged 21 years or older (5.97%). In terms of academic year, 214 participants were first-year students (53.23%) and 188 were second-year students (46.77%). The sample consisted of 56 males (13.93%) and 346 females (86.07%). This study was approved by the Ethics Committee of Neijiang Normal University (No. 2025014), and all research procedures adhered to ethical guidelines.

### Measures

**Ruminative Responses Scale (RRS).** Rumination was measured using the Ruminative Responses Scale developed by Nolen-Hoeksema and revised by Han Xiu [3]. The scale contains 22 items (e.g., “I often think about why things are going so badly for me”) and includes three dimensions: symptom rumination, compulsive thinking, and reflective pondering. All items are scored on a 4-point Likert scale from 1 (never) to 4 (always), with higher total scores representing a stronger ruminative tendency. In the present study, the Cronbach’s α for the total scale was 0.955.

**Brief Version of the Difficulties in Emotion Regulation Scale (DERS-16).** Difficulties in emotion regulation were measured with the short version of the Difficulties in Emotion Regulation Scale (DERS-16), revised by Wang [9]. The scale comprises 16 items (e.g., “When I am upset, I have difficulty controlling my behaviors”) across five dimensions, including lack of emotional clarity, difficulties engaging in goal-directed behavior, impulse control difficulties, limited access to effective emotion regulation strategies, and no acceptance of emotional responses. Items are rated on a 5-point Likert scale ranging from 1 (never) to 5 (always), with higher scores indicating greater difficulties in emotion regulation. In the present study, the Cronbach’s α for the scale was 0.935.

**Problematic Social Media Use Questionnaire.** Problematic social media use was measured using the Problematic Mobile Social Media Usage Assessment Questionnaire developed by Jiang [13]. The questionnaire contains 20 items (e.g., “Long periods of swiping on my phone often make my finger muscles sore”). Each item is rated on a 5-point Likert scale from 0 (rarely) to 4 (often), with higher scores indicating more serious problematic social media use. The scale comprises five dimensions, including increased viscosity increase, physiological damage, omission anxiety, cognitive failure, and guilt. In this study, the Cronbach’s α for the total scale was 0.924.

**Subjective Well-Being Scale.** Subjective well-being was measured using the Satisfaction with Life Scale (SWLS) and the Positive and Negative Affect Schedule (PANAS). The index of subjective well-being was calculated by adding the life satisfaction score and the positive affect score and then subtracting the negative affect score. The questionnaire has shown good applicability among Chinese samples [23]. The SWLS, developed by Diener et al. [24], consists of five items (e.g., “I am satisfied with my life”) rated on a 7-point Likert scale, with higher total scores indicating greater life satisfaction. The PANAS was developed by Watson et al. [25] and revised by Huang et al. [26], comprising two subscales, positive affect and negative affect, with a total of 20 items (e.g., “enthusiastic,” “upset”). Items are rated on a 5-point Likert scale ranging from 1 (very slightly or not at all) to 5 (extremely). Higher positive affect scores and lower negative affect scores indicate a higher level of emotional well-being. In the present study, Cronbach’s α coefficients for the SWLS and the PANAS were 0.735 and 0.885, respectively.

### Data analysis

Descriptive statistics, correlation analysis, and regression analysis were conducted using IBM SPSS 23.0. Confirmatory factor analysis and structural equation modeling were conducted using IBM SPSS AMOS 23.0.

## Results

### Test of common method bias

Harman’s single-factor test was conducted to examine potential common method bias in the measurement scales used in this study. The results showed that 14 factors had eigenvalues greater than 1, and the first factor accounted for 29.12% of the variance, which was below the recommended threshold of 40% [27]. These findings suggest that common method bias was unlikely to substantially affect the results of the present study.

### Measurement model

A confirmatory factor analysis (CFA) was conducted to evaluate the measurement model. Because the original scales contained a relatively large number of items, subscale scores were used as parcel indicators for the latent constructs. The measurement model demonstrated a good fit to the data: *χ²/df* = 2.41, GFI = 0.943, CFI = 0.976, TLI = 0.969, and RMSEA = 0.059.

As shown in Table 1, all standardized factor loadings were significant and ranged from 0.629 to 0.947. Composite reliability (CR) values ranged from 0.841 to 0.930, and average variance extracted (AVE) values ranged from 0.571 to 0.816, indicating satisfactory convergent validity and construct reliability.

**Table 1:** Factor loadings, CR, and AVE of the measurement model.

Construct	Indicator	Factor loading	SMC	CR	AVE
Rumination	Symptom rumination	0.947	0.896	0.930	0.816
	Compulsive thinking	0.878	0.771		
	Reflective pondering	0.884	0.781		
Difficulties in Emotion Regulation	Lack of emotional clarity	0.850	0.722	0.898	0.642
	Difficulties engaging in goal-Directed behavior	0.793	0.629		
	Nonacceptance of emotional responses	0.796	0.634		
	Impulse control difficulties	0.911	0.83		
	Limited access to effective emotion regulation strategies	0.629	0.395		
Problematic Social Media Use	Viscosity increase	0.670	0.449	0.841	0.571
	Physiological damage	0.750	0.562		
	Omission anxiety	0.846	0.716		
	Cognitive failure	0.746	0.557		
	Guilt	0.678	0.459		

### Descriptive and correlation analyses

Both descriptive and correlation analyses were conducted on the four variables, as given in Table 2. Results showed that rumination, difficulties in emotion regulation, and problematic social media use were all significantly negatively correlated with subjective well-being (*ps.* < 0.001). Rumination, difficulties in emotion regulation, and problematic social media use were significantly positively correlated with each other (*ps.* < 0.001). Given the gender imbalance in the sample, supplementary analyses were conducted to examine potential gender differences in the main study variables. Correlation analyses indicated that gender was not significantly associated with rumination, emotion regulation difficulties, problematic social media use, or subjective well-being (*ps.* > 0.05). Independent-samples t-tests further showed no significant gender differences in rumination, *t*(400) = −0.961, *p* = 0.337, difficulties in emotion regula*t*ion, *t*(400*)* = −0.950, *p* = 0.343, problematic social media use, *t*(400) = 0.094, *p* = 0.925, or subjec*t*ive well-being, *t*(68.83) = 0.926, *p* = 0.358. Therefore, gender was no*t* included as a control variable in subsequen*t* analyses.

**Table 2:** Descriptive Statistics and Analysis of the Correlations between Variables.

	M ± SD	1	2	3	4
1 Rumination	40.95 ± 10.97				
2 Difficulties in emotion regulation	55.63 ± 13.96	0.598^***^			
3 Problematic social media use	30.06 ± 10.45	0.732^***^	0.569^***^		
4 Subjective well-being	22.73 ± 6.89	−0.494^***^	−0.480^***^	−0.503^***^	
5 Gender		0.048	0.047	−0.005	−0.052

### Chain mediation effect test of difficulties in emotion regulation and problematic social media use

As shown in Table 3, difficulties in emotion regulation (*β* = −0.230, *p* < 0.01) and problematic social media use (*β* = −0.270, *p* < 0.001) were both negatively associated with subjective well-being. Rumination was positively associated with difficulties in emotion regulation (*β* = 0.787, *p* < 0.001) and problematic social media use (*β* = 0.432, *p* < 0.001). Difficulties in emotion regulation were positively associated with problematic social media use (*β* = 0.294, *p* < 0.001). However, the direct path between rumination and subjective well-being was not statistically significant (*β* = −0.152, *p* = 0.061).

**Table 3:** Path coefficients of the structural model.

Effect Types	B	*β*	S.E.	C.R.	*p*
Rumination → Difficulties in emotion regulation	0.684	0.787	0.041	16.534	<0.001
Rumination → Problematic social media use	0.537	0.423	0.104	5.155	<0.001
Difficulties in emotion regulation → Problematic social media use	0.428	0.294	0.118	3.636	<0.001
Difficulties in emotion regulation → Subjective well-being	−0.808	−0.230	0.280	−2.888	0.004
Problematic social media use → Subjective well-being	−0.651	−0.270	0.159	−4.089	<0.001
Rumination → Subjective well-being	−0.465	−0.152	0.248	−1.872	0.061

**Fig 1:**
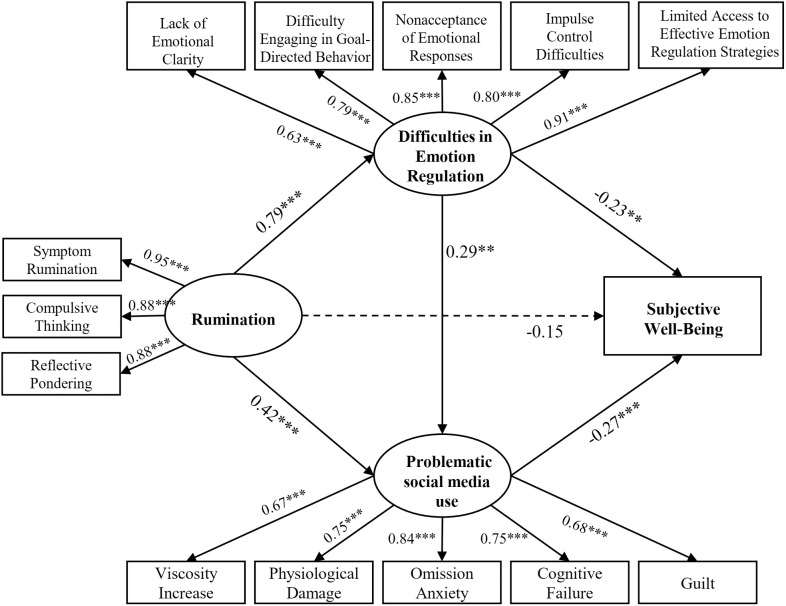
Chain mediating model (N = 402).

As shown in Fig 1, a serial mediation model was tested to examine the mediating roles of difficulties in emotion regulation and problematic social media use in the association between rumination and subjective well-being. Mediation analyses were conducted using a bias-corrected bootstrap method with 5,000 resamples to estimate 95% confidence intervals (CIs) [28]. As presented in Table 4, the 95% CIs for the three indirect paths (Ind1, Ind2, and Ind3) do not include zero, indicating that all three mediating paths were statistically significant.

**Table 4:** Results of Indirect Effect Analysis of Model.

	Estimate	Boot SE	Bootstrap 95%CI	Proportion of Mediation Effect (%)
Ind1: Rumination→Difficulties in emotion regulation→Subjective well-being	−0.181	0.061	[-0.305, -0.066]	35.490%
Ind2: Rumination→Problematic social media use→Subjective Well-Being	−0.114	0.035	[-0.195, -0.055]	22.353%
Ind3: Rumination→Difficulties in emotion regulation→Problematic social media use→Subjective well-being	−0.062	0.026	[-0.129, -0.023]	12.157%
Total mediation effect: Ind1 + Ind2 + Ind3	−0.358	0.063	[-0.488, -0.242]	70.196%
Contrast indirect effects1: Ind1 - Ind2	−0.067	0.077	[-0.218, 0.082]	
Contrast indirect effects2: Ind1 - Ind3	−0.119	0.074	[-0.260, 0.029]	
Contrast indirect effects3: Ind2 - Ind3	−0.052	0.042	[-0.151, 0.018]	

The model indexes of Fig 1 are *χ²/df* = 2.504; *p* < 0.001; GFI = 0.937; CFI = 0.971; TLI = 0.964; RMSEA = 0.061. The findings confirm that the model fits the data well, supporting the hypothesized mediation model. As shown in Table 3, difficulties in emotion regulation (95% CI = [−0.305, −0.066]) and problematic social media use (95% CI = [−0.195, −0.055]) showed significant independent indirect effects in the association between rumination and subjective well-being, accounting for 35.490% and 22.353% of the total effect, respectively. The serial indirect effect through difficulties in emotion regulation and problematic social media use was also significant (95% CI = [−0.129, −0.023]), accounting for 12.157% of the total effect. Difficulties in emotion regulation and problematic social media use also had a serial mediating effect between the independent and dependent variables (95% CI = [−0.129, −0.023]), with a mediating effect of 12.157%. Overall, the total indirect effect accounted for 70.196% of the total effect.

## Discussion

This study constructed and tested a serial mediation model examining the associations among rumination, difficulties in emotion regulation, problematic social media use, and subjective well-being based on the compensatory internet use model and the I-PACE theoretical framework. The results showed that the direct association between rumination and subjective well-being was not significant, whereas the indirect associations through difficulties in emotion regulation and problematic social media use were significant. These findings suggest that rumination, as a relatively stable cognitive trait [29], may be associated with subjective well-being primarily via emotional and behavioral intermediary mechanisms rather than through a direct path. Specifically, rumination may be related to lower subjective well-being through its associations with greater emotion regulation difficulties and maladaptive behavioral coping patterns.

At the emotional level, difficulties in emotion regulation significantly mediated the association between rumination and subjective well-being. Consistent with previous research, rumination was positively associated with difficulties in emotion regulation [10,11], whereas difficulties in emotion regulation were negatively associated with subjective well-being [4,12]. These results are consistent with a core assumption of emotion regulation theory, suggesting that rumination may be associated with reduced capacities to become aware of, understand, and accept emotional experiences, which may further relate to greater difficulties in emotion regulation and lower well-being. Individuals with higher levels of rumination tend to focus more on negative emotions and their perceived causes [30], which may further relate to greater difficulties in emotion regulation and lower subjective well-being.

At the behavioral level, problematic social media use was significantly associated with the link between rumination and subjective well-being. Consistent with prior findings, rumination was positively associated with problematic social media use [18], whereas problematic social media use was negatively associated with subjective well-being [19]. Rumination may lead vocational college students to persistently engage in negative cognition and emotions processing, which may increase psychological distress and reduce their capacity to effectively cope with real-life challenges. Under such circumstances, social media, characterized by its accessibility and immediate feedback, may be more frequently used as a strategy for stress avoidance and emotional compensation. This pattern may be associated with higher levels of problematic social media use. However, problematic social media use may be associated with temporary emotional relief, while also being related to negative outcomes in psychological and behavioral functioning. It may be associated with poorer real-life social functioning, greater upward social comparison, and lower self-control. Over time, this can harm subjective well-being [31]. In addition, this study found that rumination was not only directly associated with problematic social media use, but also indirectly associated with it through difficulties in emotion regulation. This suggests that vocational college students experiencing greater difficulties in regulating negative emotions may also report more frequent use of social media for temporary emotional relief [15]. However, such avoidance-oriented coping patterns may also be associated with stronger reliance on social media and higher levels of problematic social media use [32,33].

In addition, this study found that difficulties in emotion regulation and problematic social media use were significantly associated with the relationship between rumination and subjective well-being in the serial mediation model, whereas the direct association between rumination and subjective well-being was not significant. These findings suggest that rumination, as a cognitive vulnerability factor, may be associated with subjective well-being indirectly through emotional and behavioral correlates rather than through a direct association. Specifically, previous studies have shown that rumination is characterized by persistent focus on negative emotions and is negatively associated with adaptive emotion regulation strategies, including acceptance and cognitive reappraisal [8]. Consequently, individuals with higher levels of rumination may also report greater difficulties in emotion regulation. According to the I-PACE model, individuals with insufficient emotion regulation resources may be more likely to engage in online activities with immediate reward and emotional escape characteristics as compensatory coping strategies. Due to their accessibility, instant feedback, and attentional appeal, social media platforms may be repeatedly used in attempts to alleviate negative emotions and may be associated with higher levels of problematic social media use [15,18]. However, although such avoidance- and compensation-oriented coping strategies may provide temporary emotional relief, they may also be associated with lower levels of subjective well-being.

The present findings are generally consistent with the cognition-emotion-behavior framework proposed in the I-PACE model [20], suggesting that cognitive vulnerability, emotion regulation difficulties, and maladaptive behavioral coping patterns may be interrelated in the context of subjective well-being. Taken together, these findings highlight the importance of emotional and behavioral correlates in understanding the association between rumination and subjective well-being.

## Conclusion

This study found that difficulties in emotion regulation and problematic social media use were significantly associated with the relationship between rumination and subjective well-being through a serial mediation model. Specifically, difficulties in emotion regulation may be related to the emotional processes underlying the association between rumination and subjective well-being, whereas problematic social media use may reflect a maladaptive behavioral coping pattern associated with lower subjective well-being.

## Limitations and future directions

This study also has some limitations. First, this research mainly focused on the risk-related psychological factors and did not consider psychological resource variables that may serve protective or buffering functions. Previous research has shown that positive psychological resources, such as mindfulness [34,35], self-control [36], and self-compassion, can effectively attenuate the detrimental effects of rumination and problematic social media use on psychological functioning. Future studies could incorporate these protective factors to provide a more comprehensive understanding of how rumination relates to subjective well-being. Second, this study used a cross-sectional design, and all variables were measured using self-report scales. As a result, the findings cannot establish causal relationships or temporal dynamics among the variables, and methodological bias related to social desirability may still exist. Future research could adopt longitudinal or experimental designs to further examine the causal directions and long-term effects of the proposed model, while incorporating multi-source data to improve the objectivity of the findings. Third, the representativeness of the sample warrants cautious consideration. Participants were recruited from a single vocational college in Sichuan Province, and female students constituted the majority of the sample, which may limit the generalizability of the findings. Therefore, the results should be interpreted within this specific student population rather than generalized to all Chinese college students. Although supplementary analyses revealed no significant gender differences in the main study variables, future research should recruit more diverse and gender-balanced samples across multiple regions and institution types to further examine the robustness and external validity of the proposed model. Finally, previous research has suggested that negative emotions and problematic social media use may exhibit bidirectional associations [37]. Future studies should further examine these potential reciprocal relationships using stronger research designs.

## Supporting information

S1 Data Minimal dataset. (XLSX)

## References

[1] Diener E, Sapyta JJ, Suh E. Subjective well-being is essential to well-being. Psychol Inq. 1998;9(1):33–7. doi: 10.1207/s15327965pli0901_3

[2] Nolen-Hoeksema S, Wisco BE, Lyubomirsky S. Rethinking Rumination. Perspect Psychol Sci. 2008;3(5):400–24. doi: 10.1111/j.1745-6924.2008.00088.x 26158958

[3] Han X, Yang H. Chinese version of the Nolen-Hoeksema Ruminative Responses Scale (RRS). Chinese Journal of Clinical Psychology. 2009;17(5):550–1.

[4] Martínez-Líbano J, Axel Kosch Serey, Guillermo Barahona-Fuentes. Emotional Regulation and Subjective Well-Being in Adolescents: A Systematic Review. MHGCJ. 2025;8(1):14–26. doi: 10.56508/mhgcj.v8i1.240

[5] Wang H, Wang J, Li Z, Sun H, Zhong T, Zheng J, et al. The relationship between insomnia and non-suicidal self-injury(NSSI) among college students: parallel mediation effect of subjective well-being and rumination. BMC Psychol. 2025;13(1):969. doi: 10.1186/s40359-025-03335-2 40859317 PMC12382126

[6] Chen Q, Wei L, Luo X. Research on the influence of negative life events on the subjective well-being of students of preschool major in vocational colleges and the chain mediation effect. Journal of Shaanxi Xueqian Normal University. 2021;37(12):92–7. doi: 10.11995/j.issn.2095-770X.2021.12.013

[7] Peng H, Sheng L, Qiu F. Psychological burden reduction starts from dealing with boredom: The influencing mechanism of boredom proneness on subjective well-being among adolescents. Psychological Development and Education. 2023;39(6):895–902. doi: 10.16187/j.cnki.issn1001-4918.2023.06.16

[8] Aldao A, Nolen-Hoeksema S, Schweizer S. Emotion-regulation strategies across psychopathology: A meta-analytic review. Clin Psychol Rev. 2010;30(2):217–37. doi: 10.1016/j.cpr.2009.11.004 20015584

[9] Wang G, Guo W, Shen JJ. Validity and reliability of the brief version of the difficulties in emotion regulation scale in Chinese college students. Chinese Journal of Clinical Psychology. 2021;29(5):956–61. doi: 10.16128/j.cnki.1005-3611.2021.05.013

[10] Mansueto G, Marino C, Palmieri S, Offredi A, Sarracino D, Sassaroli S, et al. Difficulties in emotion regulation: The role of repetitive negative thinking and metacognitive beliefs. J Affect Disord. 2022;308:473–83. doi: 10.1016/j.jad.2022.04.086 35460736

[11] Chen J, Fang X, Zhang K. Effect of attachment anxiety on nomophobia in college students: Chain mediating effect. China Journal of Health Psychology. 2023;31(11):1747–52. doi: 10.13342/j.cnki.cjhp.2023.11.027

[12] Zhuo R. The impact of impulsivity on subjective well-being of college freshmen: A chain mediation model. The Guide of Science & Education. 2025(16):82–4. doi: 10.16400/j.cnki.kjdk.2025.16.027

[13] Jiang Y. Development of a problematic mobile social media usage assessment questionnaire for adolescents. Psychology: Techniques and Applications. 2018;6(10):613–21. doi: 10.16842/j.cnki.issn2095-5588.2018.10.004

[14] Kardefelt-Winther D. A conceptual and methodological critique of internet addiction research: Towards a model of compensatory internet use. Computers in Human Behavior. 2014;31:351–4. doi: 10.1016/j.chb.2013.10.059

[15] Chentsova VO, Bravo AJ, Mezquita L, Pilatti A, Hogarth L, Cross-Cultural Addictions Study Team. Internalizing symptoms, rumination, and problematic social networking site use: A cross national examination among young adults in seven countries. Addict Behav. 2023;136:107464. doi: 10.1016/j.addbeh.2022.107464 36067636

[16] Chen Y, Zhang Y, Zhang S. Effect of fear of missing out on college student’s negative social adaptation: Chain-mediating effect of rumination and problematic social media use. China Journal of Health Psychology. 2022;30(4):581–6. doi: 10.13342/j.cnki.cjhp.2022.04.021

[17] Chu Y, Jiang Y, Mao Z. Stress perception and problematic smartphone use in adolescents: The role of rumination and emotion regulation. Psychology: Techniques and Applications. 2022;10(12):731–9. doi: 10.16842/j.cnki.issn2095-5588.2022.12.004

[18] Sam I. The mediating role of rumination and emotion regulation on the relationship between perceived stress and problematic smartphone use among adolescents. Psycho-Educational Research Reviews. 2024;13(3):159–68. doi: 10.52963/PERR_Biruni_V13.N3.03

[19] Zhao L. The association between social media use intensity, problematic use, and subjective well-being of users: A comparative study of young and older users. Population and Society. 2022;38(6):94–104. doi: 10.14132/j.2095-7963.2022.06.008

[20] Brand M, Young KS, Laier C, Wölfling K, Potenza MN. Integrating psychological and neurobiological considerations regarding the development and maintenance of specific Internet-use disorders: An Interaction of Person-Affect-Cognition-Execution (I-PACE) model. Neurosci Biobehav Rev. 2016;71:252–66. doi: 10.1016/j.neubiorev.2016.08.033 27590829

[21] López-Montón M, Aonso-Diego G, Estévez A. Emotional Distress and Body Dissatisfaction: The Mediating Role of Social Media and Emotional Regulation. Behav Sci (Basel). 2024;14(7):580. doi: 10.3390/bs14070580 39062403 PMC11274327

[22] Brown DJ, Scott R, Ireland R, Harness J, Phipps DJ, Keech JJ. Rethinking social media and mental health: The role of emotion regulation difficulties. Computers in Human Behavior. 2026;174:108825. doi: 10.1016/j.chb.2025.108825

[23] Hu Y, He Z, Zeng Z. A longitudinal relationship between body surveillance and depressive symptoms among junior school students: The mediating role of subjective well-being. Studies of Psychology and Behavior. 2025;23(1):58–65. doi: 10.12139/j.1672-0628.2025.01.008

[24] Diener E, Emmons RA, Larsen RJ, Griffin S. The Satisfaction With Life Scale. J Pers Assess. 1985;49(1):71–5. doi: 10.1207/s15327752jpa4901_13 16367493

[25] Watson D, Clark LA, Tellegen A. Development and validation of brief measures of positive and negative affect: the PANAS scales. J Pers Soc Psychol. 1988;54(6):1063–70. doi: 10.1037//0022-3514.54.6.1063 3397865

[26] Huang L, Yang T, Ji Z. Applicability of the positive and negative affect scale in Chinese. Chinese Mental Health Journal. 2003;1:54–6.

[27] Zhou H, Long L. Statistical remedies for common method biases. Advances in Psychological Science. 2004(6):942–50. doi: 10.3969/j.issn.1671-3710.2004.06.018

[28] Wen Z, Ye B. Different Methods for Testing Moderated Mediation Models: Competitors or Backups?. Acta Psychologica Sinica. 2014;46(5):714. doi: 10.3724/sp.j.1041.2014.00714

[29] Shaw ZA, Hilt LM, Starr LR. The developmental origins of ruminative response style: An integrative review. Clin Psychol Rev. 2019;74:101780. doi: 10.1016/j.cpr.2019.101780 31739123

[30] Zhou Y, Cai W. Relationship between adjustment disorder and suicidal ideation in newly employed / enrolled young adults and mediating effects of rumination and negative emotions. Journal of Zhengzhou University (Medical Sciences). 2025;60(2):265–9. doi: 10.13705/j.issn.1671-6825.2024.05.040

[31] Li M, Khairani AZ, Jiang W. The impact of problematic social media use on subjective well-being among higher vocational college students: The chain mediating role of psychological distress and sleep disturbance. Medicine (Baltimore). 2025;104(24):e42542. doi: 10.1097/MD.0000000000042542 40527840 PMC12173272

[32] Lin M-P. Avoidance/emotion-focused coping mediates the relationship between distress tolerance and problematic Internet use in a representative sample of adolescents in Taiwan: One-year follow-up. J Adolesc. 2022;94(4):600–10. doi: 10.1002/jad.12049 35403220

[33] Chen X, Peng S, Guan H, Sun H, Wu H, Yao X, et al. Effect of emotional intelligence on problematic mobile social media use: mediating role of peer relationships and experiential avoidance. Front Psychol. 2025;16:1558733. doi: 10.3389/fpsyg.2025.1558733 40612993 PMC12222163

[34] van Seggelen-Damen ICM, Peeters SCT, Jacobs N. Being mindful and resilient: The role of self-reflection, rumination, and well-being. Psychology of Consciousness: Theory, Research, and Practice. 2023;10(2):193–203. doi: 10.1037/cns0000338

[35] Meynadier J, Malouff JM, Loi NM. Lower mindfulness is associated with problematic social media use: A meta-analysis. Current Psychology. 2024;43(4):3395–404. doi: 10.1007/s12144-023-04587-0

[36] Du J, Kerkhof P, van Koningsbruggen GM. The reciprocal relationships between social media self-control failure, mindfulness and wellbeing: A longitudinal study. PLoS One. 2021;16(8):e0255648. doi: 10.1371/journal.pone.0255648 34347832 PMC8336798

[37] Shen G, Huang G, Wang M, Jian W, Pan H, Dai Z, et al. The longitudinal relationships between problematic mobile phone use symptoms and negative emotions: a cross-lagged panel network analysis. Compr Psychiatry. 2024;135:152530. doi: 10.1016/j.comppsych.2024.152530 39303373

